# Elemental Profiles in *Cycas micronesica* Stems

**DOI:** 10.3390/plants7040094

**Published:** 2018-11-01

**Authors:** Thomas E. Marler

**Affiliations:** College of Natural and Applied Sciences, University of Guam, UOG Station, Mangilao, Guam 96923, USA; thomas.marler@gmail.com; Tel.: +1-671-735-2100

**Keywords:** cycad, gymnosperm, nutrient concentration, stoichiometry

## Abstract

Essential nutrients and metals have been quantified in stems of many tree species to understand the role of stems as storage and source organs. Little is known about stored stem resources of cycad tree species. *Cycas micronesica* tissue was collected from apical and basal axial regions of stems; and pith, vascular, and cortex tissues were separated into three radial regions. Leaves were also sampled to provide a comparison to stems. Minerals and metals were quantified in all tissues. Minerals and metals varied greatly among the six stem sections. Phosphorus varied more among the three radial sections than the other macronutrients, and zinc and nickel varied more than the other micronutrients. Stem carbon was less than and stem calcium was greater than expected, based on what is currently known tree stem concentrations in the literature. Elemental concentrations were generally greater than those previously reported for coniferous gymnosperm trees. Moreover, the stem concentrations were high in relation to leaf concentrations, when compared to published angiosperm and conifer data. The results indicated that the addition of more cycad species to the literature will improve our understanding of gymnosperm versus angiosperm stem nutrient relations, and that the non-woody cycad stem contains copious essential plant nutrients that can be mobilized and deployed to sinks when needed.

## 1. Introduction

The study of biogeochemistry requires valuations of allocation, resorption, sequestration, and translocation of minerals and metals among all plant organs [1,2,3]. This elemental composition of various tissues within a plant is referred to as the ionome [4,5]. The component of the ionome that has been most studied is the essential nutrient composition of the active photosynthetic leaf [6]. However, various organs and tissues within a plant perform highly contrasting functions, and plants adjust the allocations of elements among these organs to maximize organ function and overall plant growth [7,8]. Moreover, the approach that plants employ to allocate available components of the ionome among competing organs is crucial for adapting to environmental stresses [9,10].

The reports on tree stem elemental concentrations have largely focused on woody tree growth forms. In general, leaf concentrations of essential minerals exceed those of woody stems, and the outer active annulus of stems contains greater concentrations than the inner core [7,10,11,12,13,14]. Furthermore, gymnosperms as a group contain less nutrients in stems than angiosperms [11].

**Figure 1:**
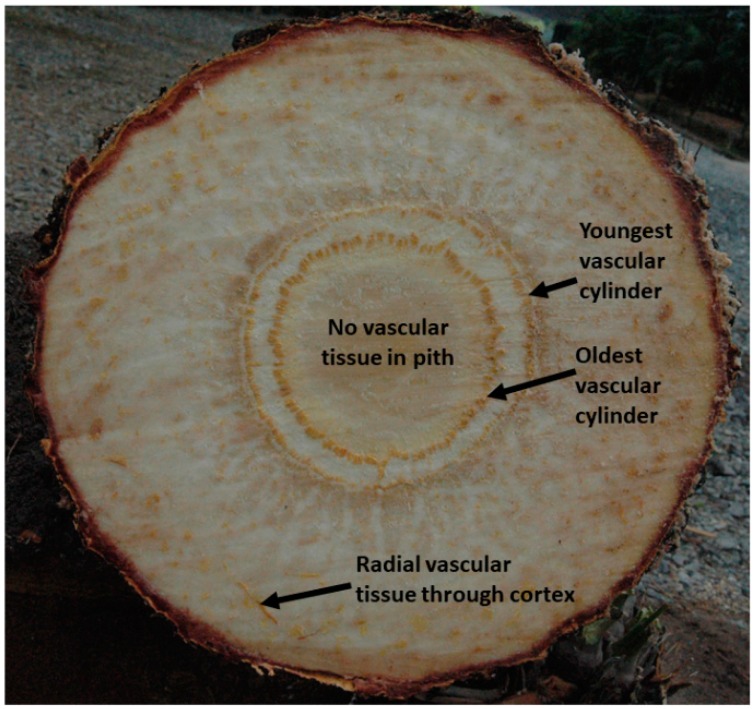
Arborescent cycad plant stems are comprised of central persistent parenchymatous pith, concentric vascular cylinders that connect roots to stem apex, and persistent peripheral parenchymatous cortex. Radial vascular stands pass through cortex to connect the vascular cylinders to leaves. This vascular tissue persists in the cortex after leaf senescence.

One plant group that has been noticeably missing from this research agenda is cycads. Contemporary cycads are gymnosperms and are comprised of ca. 350 described species [15]. Cycads encompass the most threatened group of plants worldwide [16,17]. Conservation of cycads is of interest in part because of their unique evolutionary history, importance in evolution of entomophily [18], and position in present-day phylogenetic diversity [18,19,20,21]. The cycad stem does not contain a typical secondary vascular cambium layer, and its large primary thickening meristem creates a pachycaulous growth habit [18]. The outer cortex and inner pith are parenchymatous and retained for life (Figure 1). The vascular tissue that is located between these two radial sections also contains copious parenchyma tissue. These stem traits predict that the ionome of a cycad stem will not conform to the stem ionome described in the literature for arborescent species [7,10,11,12,13,14].

*Cycas micronesica* (*C. micronesica*) K.D. Hill is an arborescent cycad species from several island groups in Micronesia [22]. This tree is threatened with extinction due to the recent invasions of several specialist insect pests [23,24]. My objective was to add a cycad species to the stem ionomics literature by determining the profile of essential elements and non-essential metals within *C. micronesica* stems among three radial zones and two axial zones. Inclusion of leaf measurements enabled a comparison of leaf versus stem concentrations for a range of elements.

## 2. Materials and Methods 

Ten healthy container-grown *C. micronesica* plants were used in July 2016 to obtain tissue samples from six stem locations. The plants were grown from seeds collected from Eastern Guam and then planted in a nursery at the University of Guam in 2010. The initial container size was 2.6 L, and the peat:perlite ratio of initial container medium was 60:40. The plants were transferred to larger containers as needed such that the final container size was 15.5 L. The container medium after the first transplant had equal parts of peat, perlite, sand and field soil. The plants were grown underneath 50% shade screen for the first 3 years, and under full sun thereafter. Rainfall was augmented with hand-watering as needed. A granular fertilizer (12N–2.6P–6.6K plus micronutrients) was provided to each container approximately every 8 weeks at the rate of 5 g initially and 30 g for the final container size. In addition, a 125-mL drench of 2.5 mM Fe (as Fe-EDDHA) solution was applied to each container every 8 weeks. Protection from armored scale infestations was provided by root application of imidacloprid every 2–3 months. The total plant height ranged from 155 cm to 188 cm, and the stem height ranged from 111 cm to 122 cm. Plants with this height exhibit linear pachycaulous stem dimensions.

Leaf chemistry was characterized by collecting leaflets from the apical, midpoint, and basal area of every leaf on each plant, and then combining them into one homogenized sample for each replication. This ensured every leaf was represented, regardless of age. A 1-cm disc was cut from each stem directly below the leaves. A second 1-cm disc was cut from the base of the stems immediately above the root collar. These two axial positions will be referred to as apical and basal hereinafter. Each disc was separated into three radial sections (Figure 1). The peripheral section was the cortex, the intermediate section was comprised of the concentric cylinders of axial vascular tissue, and the innermost section was the pith. As cycad trees increase in age and stem diameter, the increase in diameter is constricted to the vascular cylinder section. There is no ambiguity in the demarcations between each of these three radial sections, and they will be referred to as cortex, vascular, and pith sections hereinafter. The unused portion of the stems were used for asexual propagation of each plant.

The leaf tissue and the tissue from the six stem positions was dried at 75 °C and milled to pass through a 20-mesh screen. Total nitrogen and carbon were determined by dry combustion (FLASH EA1112 CHN Analyzer, Thermo Fisher, Waltham, MA, USA). Samples were digested by a microwave system with nitric acid and peroxide, and then other essential elements and metals were quantified by inductively coupled plasma optical emission spectroscopy (Spectro Genesis; SPECTRO Analytical Instruments, Kleve, Germany) [25]. Chlorine, sulfur, and molybdenum were not included because the established protocol for the agricultural industries in the region do not include these two elements.

The statistical analysis of the stem locations was conducted using SAS 9.3 (SAS Institute, Cary, Indiana), employing a 2 × 3 factorial statistical analysis with two axial positions (apical and basal) and three radial positions (cortex, vascular, pith), as described previously [26]. Means separation was conducted by Least Significant Difference for response variables that exhibited significance. The percentage of radial discrepancy was determined for each element by ranking pith, vascular, and cortex in order and then calculation was performed with the equation: ((maximum − minimum)/maximum)) × 100. The leaf results were not included in a statistical procedure, but were used to determine stem concentrations in relation to leaf concentrations by the leaf/stem ratio. For this metric, the overall mean of the six stem locations was used as the denominator.

## 3. Results

### 3.1. Radial Differences in Resource Allocation

Six of the response variables did not exhibit an interaction between radial and axial *C. micronesica* stem sections but differed significantly among the radial tissue sections (Table 1). The vascular tissues contained more carbon and iron than the other tissues, and both elements did not differ between pith and cortex tissues. Phosphorus and lead concentrations were greatest in cortex tissues. Zinc and arsenic concentrations were greatest in pith tissues.

### 3.2. Interactions among Axial and Radial Stem Sections

Eight of the response variables exhibited concentrations that behaved differently among the six sampled *C. micronesica* stem sections, generating a significant interaction (Table 2). Nitrogen was greatest within cortex tissue in the apical region, but did not differ among radial sections in the basal region. Potassium, magnesium, and boron were greatest within vascular tissue in the apical region, but did not differ among radial sections in the basal region. Patterns of sodium, cobalt, chromium, and nickel were unique.

### 3.3. Axial Differences in Element Concentrations

Three of the elements did not exhibit an interaction between axial and radial *C. micronesica* stem locations, but exhibited significantly greater concentrations in the apical position than in the basal position (Table 3). The elemental concentrations from the base of the stems ranged from 8% to 56% of those from the apex of the stems.

### 3.4. Unaffected Elements

With a mean of 15.15 mg·g^−1^, calcium concentration was not influenced by axial or radial positions in the *C. micronesica* stems. Cadmium (mean = 0.03 μg·g^−1^) and selenium (mean = 0.73 μg·g^−1^) concentrations were also not influenced by the stem position.

### 3.5. Leaf Tissue

Carbon concentrations greatly exceeded the concentrations of all minerals and metals in the *C. micronesica* leaves (Table 4). Macronutrient concentrations of green leaves were within expected ranges and followed the order: nitrogen > potassium > phosphorus = calcium > magnesium. Micronutrient concentrations were also as expected and followed the order: iron > manganese > zinc > boron > copper > nickel. Concentrations of the non-essential metals—lead, selenium, and chromium—exceeded concentrations of some of the essential micronutrients.

### 3.6. Derived Traits

Half of the elements were accumulated in greater quantities in leaves than in stems (leaf:stem ratio > 1.0, Table 5). Leaf nitrogen, copper, and cadmium concentrations were more than twice those of stems. Half of the elements were accumulated in greater quantities in stems than in leaves (leaf:stem ratio < 1.0). Stem calcium, sodium, arsenic, and lead concentrations were more than twice those of leaves.

The radial discrepancy of the *C. micronesica* stems varied greatly among the elements (Table 5). Phosphorus varied more radially and carbon varied less radially than the other macronutrients. Zinc and nickel were the micronutrients that exhibited the greatest radial discrepancy, and copper was the micronutrient that exhibited the least radial discrepancy. Among the non-essential metals, lead exhibited the greatest radial discrepancy and cadmium exhibited the least radial discrepancy.

## 4. Discussion

Applied research into structure and function of the unique cycad stem has been deficient. The pachycaulous cycad stem is comprised of persistent peripheral cortex and central pith tissues, and these two radial sections are separated by concentric vascular cylinders (see images in [25,27,28]). The parenchymatous cortex is not replaced by bark, but a wound periderm is rapidly formed, following wounding to seal off damaged tissue with phellem [28]. Radial hydraulic connections occur through the vascular cylinders and vascular traces through the cortex connect leaves with the vascular cylinders [28]. The scaling of the stem height to the basal stem diameter is unique for pachycaulous species as a group [29]. Using *C. micronesica* as an example of pachycaulous arborescent cycads, female plants exhibited greater height per increment diameter increase than male plants [30]. When *C. micronesica* stems are inclined, asymmetric tissue growth occurs on the lower side of the vascular tissues but no compression wood is exhibited in this asymmetric growth [31,32]. The relative proportion of each radial zone is highly contrasting among *Cycas* species, and the relative width of the cortex for a given species is correlated with relative ease of horticultural care [27]. Radial differences in non-structural carbohydrates of *C. micronesica* stems are substantial, but axial differences are minimal [26]. Large stem cuttings of *C. micronesica* are capable of developing adventitious roots for successful asexual propagation, and depletion of stem carbohydrates, however, following pest infestations compromises this capability [33].

Herein, I extend this limited knowledge by reporting, for the first time, cycad stem contents of a range of essential nutrients and non-essential metals. The results indicated that stems of this arborescent cycad species contain greater concentrations of macronutrients than those of other gymnosperms that have been studied [11]. The claims that gymnosperms have lower mineral nutrient concentrations in stems than angiosperms; therefore, it is not valid until more trees within the Cycadophyta and Ginkgophyta divisions are added to the global dataset. As argued elsewhere [34], when conifers are used and the other divisions of gymnosperms are not included in a dataset, the division Coniferophyta should be used to describe the phylogenetic grouping rather than gymnosperm. Elemental analysis of stems of other cycad species has occurred only one other time, to my knowledge [35], but unfortunately, the concentrations of the *Macrozamia riedlei* (Gaud.) C.A. Gardn. in stems were not reported even though they were measured. The relative order of concentrations of the measured elements in the stems was not the same as that of the leaves. Calcium was the main element that differed, exhibiting the second most abundant element in stems and the fifth most abundant element in leaves.

Published contrasts of stem versus leaf components are abundant for woody tree species [7,10,12,13,14]. The macronutrients other than carbon occurred in greater concentration in *C. micronesica* stems relative to *C. micronesica* leaves when compared to the literature. In other words, the relative amount of macronutrients stored in *C. micronesica* stems exceeded that stored in woody angiosperm and gymnosperm stems. Calcium behavior deviated the most from the published results, in that woody tree stems contained less calcium than leaves, and yet the stems of this cycad species contained greater calcium than the leaves. Since these copious sequestered elements reside in live stem tissues, they are presumably available for mobilization and deployment to other organs during times of rapid organ construction. This trait decreases a cycad plant’s dependence on contemporary nutrient acquisition from soils to supply sink activity.

The *C. micronesica* stems contained 39% carbon (mean from Table 1), which was less than that of leaves (Table 4). Published reports on numerous tree species indicated similar carbon concentrations for stems and leaves [36,37], so the greater value for cycad leaves is of interest. More cycad species should be studied to determine if this is a canonical cycad trait. This value for a cycad stem is also well below the 45%-to-60% range for the carbon concentration that has been reported for stems of forest tree species [36,38,39,40]. A greater understanding of the global carbon balance requires estimates of carbon storage in forest ecosystems. These estimates are crafted based on biomass estimates of land-use areas, and the equations that are employed require refined data to improve accuracy [37,41,42,43]. One unsophisticated approach is to estimate carbon sequestration based on plant biomass, using the assumption that aboveground forest biomass is 50% carbon [44]. However, considerable variations of stem carbon sink activity occur among tree species [45]. My results indicated that the use of accepted averages of important metrics like carbon content or specific gravity to estimate biomass or carbon storage would not be accurate in habitats where a cycad species is prevalent. In contrast, species-specific measurements of biomass and carbon concentrations are needed. An estimation of stem biomass for a cycad species has been conducted for *M. riedlei* [35], and the reported biomass concentration of 2531 kg·ha^−1^ reveals the role of cycads in sequestering carbon may be substantial.

The dynamics of radial variations in carbon content also differ greatly for this cycad stem versus woody stems. The dead heartwood in woody tree species contains greater carbon concentration than the peripheral live sapwood [46]. These general traits may be used to predict radial variations in stem carbon, based on the relative proportion of heartwood to sapwood for a given species. The inner core would contain greater carbon than the outer annulus in these predictive approaches. In contrast, the inner core of a cycad stem is live pith. The intermediate annulus of *C. micronesica* vascular tissue contained greater carbon than the inner pith and outer cortex annulus (Table 1). This contrasted with earlier reporting that pith tissues contained the greatest concentrations of non-structural carbohydrates [26]. Presumably, the carbon in the vascular tissues resides in structural macromolecules, which would explain the differences between carbon and non-structural carbohydrate behaviors. Moreover, as cycad trees increase in age and size, the vascular cylinders become a greater relative proportion of the stem volume. Therefore, the mean concentration of carbon in a cycad stem may increase more with plant ontogeny than that occurring with a woody tree species.

The radial differences of minerals in tree stems has garnered considerable attention [11,13,47]. Unlike carbon, the focus on minerals has been the extent of mobility as wood ages from sapwood to heartwood. The peripheral active wood acts as a sink, and the most recently added annulus of heartwood is viewed as a senescing source, where the nutrients are mobilized and moved peripherally to the sapwood. As discussed elsewhere, these analogies are impossible to apply to a cycad stem because no stem tissue senesces for the life of the plant and no discrete secondary cambium occurs [26]. Despite the lack of an analog from the literature, my results indicated that considerable radial variation occurs for many minerals and metals in this representative cycad stem as a result of the discrete tissue zones and their disparate function. Interestingly, phosphorus varied more radially than the other mineral macronutrients in woody tree species [11,13,47] and in this cycad species (Table 5).

## 5. Conclusions

The carbon and mineral relations of *C. micronesica* stems revealed several deviations from the published generalities on woody angiosperm and gymnosperm trees, with less carbon and more calcium than those expected. Many aspects of functional ecology have used scaling and allometric approaches to look at large data patterns within relationships among organs, elements, plant functional and phylogenetic groupings, latitudinal and other gradients, and more [7,10,11,12,13,14]. Most contemporary publications on cycad biology include a discussion about how cycads have been ignored in the past and ongoing large-scale research agendas, and the failure to include the cycad stem ionome in relevant research topics is no exception. Indeed, this is the first cycad species to be studied in this context. This knowledge gap should be addressed by determining more about the relationship between the stem and leaf ionome of other cycad species, so that cycads can be adequately added to the accumulating worldwide data sets.

## Figures and Tables

**Table 1:** The influence of radial position within *Cycas micronesica* stems on mineral and metals. Means are a combination of apical and basal stem regions. Data were presented as mean ± standard error for n = 20.

Variable	Pith	Vascular	Cortex	Significance
Carbon (mg·g^−1^)	379.92 ± 3.44 a ^1^	403.09 ± 2.75 b	381.18 ± 3.02 a	<0.0001
Phosphorus (mg·g^−1^)	1.69 ± 0.28 a	2.46 ± 0.26 ab	3.62 ± 0.36 b	0.0191
Iron (μg·g^−1^)	46.70 ± 3.42 a	81.51 ± 5.95 b	58.59 ± 5.20 a	<0.0001
Zinc (μg·g^−1^)	21.10 ± 5.57 b	16.24 ± 3.08 ab	8.07 ± 0.97 a	0.0414
Arsenic (μg·g^−1^)	0.34 ± 0.08 b	0.14 ± 0.05 a	0.13 ± 0.04 a	0.0021
Lead (μg·g^−1^)	2.69 ± 0.36 b	0.56 ± 0.07 a	4.22 ± 0.40 c	<0.0001

^1^ Means followed by the same letter within each row are not different according to Least Significant Difference.

**Table 2:** The influence of axial and radial locations within *Cycas micronesica* stems on various elements. Data were presented as mean ± standard error for n = 10.

Variable	Apex Pith	Apex Vascular	Apex Cortex	Base Pith	Base Vascular	Base Cortex	Significance
Nitrogen (mg·g^−1^)	11.33 ± 1.33 a^1^	11.25 ± 0.77 a	16.62 ± 1.94 b	10.46 ± 0.93 a	10.53 ± 0.73 a	10.21 ± 0.73 a	0.0332
Potassium (mg·g^−1^)	6.54 ± 0.91 a	16.27 ± 3.90 b	9.56 ± 2.24 ab	5.58 ± 1.49 a	5.26 ± 0.73 a	8.10 ± 1.61 ab	0.0329
Magnesium (mg·g^−1^)	2.56 ± 0.38 ab	4.94 ± 0.99 c	3.50 ± 0.39 bc	1.67 ± 0.11 a	0.98 ± 0.06 a	1.91 ± 0.15 a	0.0158
Sodium (mg·g^−1^)	0.98 ± 0.28 a	2.34 ± 0.57 b	1.65 ± 0.31 a	1.92 ± 0.31 ab	0.75 ± 0.15 a	2.30 ± 0.30 b	0.0088
Boron (μg·g^−1^)	9.00 ± 1.46 a	20.50 ± 3.73 b	13.37 ± 1.49 a	7.61 ± 1.64 a	9.01 ± 0.31 a	13.14 ± 0.83 a	0.0080
Cobalt (μg·g^−1^)	0.01 ± 0.00 a	0.01 ± 0.00 a	0.05 ± 0.02 b	0.01 ± 0.00 a	0.06 ± 0.01 b	0.04 ± 0.01 b	0.0245
Chromium (μg·g^−1^)	0.25 ± 0.02 a	0.27 ± 0.03 a	0.49 ± 0.09 b	0.25 ± 0.03 a	0.44 ± 0.03 b	0.30 ± 0.03 a	0.0004
Nickel (μg·g^−1^)	0.01 ± 0.00 a	0.08 ± 0.02 b	0.01 ± 0.00 a	0.11 ± 0.04 b	0.09 ± 0.01 b	0.01 ± 0.00 a	0.0478

^1^ Means followed by the same letter within each row are not different according to Least Significant Difference.

**Table 3:** The influence of axial position within *Cycas micronesica* stems on various elements. Means are combination of pith, vascular, and cortex radial stem regions. Data were presented as mean ± standard error for n = 30.

Variable	Apical	Basal	Significance
Manganese (μg·g^−1^)	21.96 ± 3.39	12.40 ± 1.16	0.0071
Copper (μg·g^−1^)	1.99 ± 0.14	0.92 ± 0.10	<0.0001
Arsenic (μg·g^−1^)	0.37 ± 0.06	0.03 ± 0.01	<0.0001

**Table 4:** Essential elements and metal concentrations in *Cycas micronesica* leaves. Data were presented as mean ± standard error for n = 10.

Element	Concentration	Element	Concentration
Carbon (mg·g^−1^)	478.9 ± 18.8	Copper (μg·g^−1^)	4.2 ± 0.2
Nitrogen (mg·g^−1^)	25.1 ± 2.5	Boron (μg·g^−1^)	13.6 ± 0.1
Potassium (mg·g^−1^)	15.3 ± 0.6	Arsenic (μg·g^−1^)	0.08 ± 0.03
Phosphorus (mg·g^−1^)	2.9 ± 0.1	Cadmium (μg·g^−1^)	0.04 ± 0.01
Calcium (mg·g^−1^)	2.8 ± 0.1	Cobalt (μg·g^−1^)	ND ^z^
Magnesium (mg·g^−1^)	2.3 ± 0.1	Chromium (μg·g^−1^)	0.28 ± 0.01
Sodium (mg·g^−1^)	0.5 ± 0.03	Nickel (μg·g^−1^)	0.04 ± 0.03
Manganese (μg·g^−1^)	23.8 ± 2.3	Lead (μg·g^−1^)	0.60 ± 0.04
Iron (μg·g^−1^)	43.5 ± 0.6	Selenium (μg·g^−1^)	0.58 ± 0.25
Zinc (μg·g^−1^)	19.0 ± 0.9		

^z^ Not detected.

**Table 5:** The calculated leaf/stem ratios of concentrations of minerals and metals and the radial discrepancy of minerals and metals in stems calculated as ((maximum − minimum)/maximum) × 100 for *Cycas micronesica* plants. Data were presented as mean ± standard error for n = 10.

Variable	Leaf:Stem Ratio	Radial Discrepancy (%)
Nitrogen	2.13	19.08
Carbon	1.23	5.75
Phosphorus	1.10	53.31
Potassium	1.79	40.35
Calcium	0.18	30.23
Magnesium	0.88	25.35
Sodium	0.31	24.75
Manganese	1.39	33.98
Iron	0.70	42.71
Zinc	1.26	61.76
Copper	2.87	16.13
Boron	1.12	40.00
Arsenic	0.38	61.76
Cadmium	2.06	9.09
Chromium	0.85	37.50
Nickel	0.80	87.50
Lead	0.24	86.73
Selenium	0.80	37.50
